# Cognitive Behavioral Therapy for Obsessive-Compulsive Disorder with Comorbid Schizophrenia: A Case Report

**DOI:** 10.1192/j.eurpsy.2025.2138

**Published:** 2025-08-26

**Authors:** S. Laabidi, O. Laabidi, U. Ouali, A. Aissa, R. Jomli

**Affiliations:** 1 psychiatry A, Razi hospital, manouba, Tunisia

## Abstract

**Introduction:**

In the general population, the lifetime prevalence rates for obsessive-compulsive disorder (OCD) range between 1.9% and 3.3%. In patients with schizophrenia, the prevalence rates of OCD range between 7.8% and 26%. Accurate diagnosis has prognostic and treatment implications.The empirical basis regarding the optimal treatment for comorbid OCD in patients with schizophrenia is almost nonexistent.

**Objectives:**

We report a successful treatment course of intensive CBT for a patient with OCD comorbid with schizophrenia and reflect on the difficulties in the management and treatment of these cases.

**Methods:**

We describe a case report in which OCD emerged gradually after the remission of positive symptoms of schizophrenia. The CBT involved psychoeducation, case formulation, cognitive restructuring, and exposure and response prevention.

**Results:**

The case is a 24-year-old male, single, with no comorbid somatic diseases. He was admitted to our psychiatric ward for self-muttering activity and delusion of thought broadcasting from the past year. A diagnosis of schizophrenia was made. We started risperidone gradually titrated to 6 mg/day. During his follow-up period, he reported having repetitive and intrusive thoughts of blasphemous nature despite well-controlled psychotic symptoms. He acknowledged these thoughts as originating in his own mind but was unable to stop them on his own accord. He also reported obsessions related to contamination and disgust. This led to compulsive hand washing and avoidance behaviour of some objects, which was both distressing and time-consuming. The diagnosis was revised to comorbid schizophrenia and OCD. Antipsychotic was changed from risperidone to amisulpride 800 mg daily in combination with paroxetine up to 60 mg/d. Since paroxetine was already optimized, the next step taken was to substitute it. He was then medicated with amisulpride, and clomipramine slowly increased up to 225 mg/d. There was no significant clinical improvement, regardless of the dose. Cognitive behavioural therapy (CBT) was commenced later. Medication was kept stable during the baseline, treatment, and follow-up period. Fourteen 1-hour sessions of CBT, including exposure and response prevention, were delivered each week over a period of 14 weeks. At the end of the intensive treatment, he reported a significant reduction in obsessions and compulsions. His score on the Y-BOCS dropped from 34 to 8 (76%) before treatment to 4-month follow-up.He reported that the decrease in OCD symptoms was associated with a significantly higher quality of life.

**Conclusions:**

CBT appears to offer a valuable opportunity to reduce symptom severity in patients with OCD comorbid with schizophrenia.Further research within this field and systematic clinical evaluations are highly desirable.

**Disclosure of Interest:**

None Declared

